# Characterization of Polysaccharide-Based Composites Enriched with Zinc Oxide and Bacitracin for the Treatment of Infected Wounds

**DOI:** 10.3390/gels12030218

**Published:** 2026-03-06

**Authors:** Alicja Macyk, Anna Kusibab, Dorota Ochońska, Monika Brzychczy-Włoch, Katarzyna Reczyńska-Kolman, Elżbieta Pamuła

**Affiliations:** 1 Department of Biomaterials and Composites, Faculty of Materials Science and Ceramics, AGH University of Krakow, Mickiewicza Avenue 30, 30-059 Kraków, Poland; amacyk@student.agh.edu.pl (A.M.); amoskwik@agh.edu.pl (A.K.); kmr@agh.edu.pl (K.R.-K.); 2 Department of Molecular Medical Microbiology, Chair of Microbiology, Faculty of Medicine, Jagiellonian University Medical College, ul Czysta 18, 31-121 Kraków, Poland; dorota.ochonska@uj.edu.pl (D.O.); m.brzychczy-wloch@uj.edu.pl (M.B.-W.)

**Keywords:** hydrogels, gellan gum, sodium alginate, zinc oxide nanoparticles, bacitracin, wound dressings

## Abstract

This study aimed to manufacture and characterize highly porous dressings based on gellan gum (GG) and sodium alginate (Alg) hydrogels modified with zinc oxide (ZnO) and bacitracin (BAC) intended for infected and exuding wounds. ZnO nanoparticles (ZnO(n)) were 26 ± 4 nm in size according to atomic force microscopy (AFM), while the size of the microparticles (ZnO(m)) was 1.02 ± 0.01 µm according to laser diffraction measurements. Their relative surface areas were 39.16 m^2^/g and 4.56 m^2^/g, respectively. Microbiological studies showed that ZnO(n) exhibited antibacterial activity in contact with the Gram+ *Staphylococcus aureus*; thus, they were selected for embedding in a hydrogel matrix. Four types of composite hydrogel samples were manufactured: GG/Alg, GG/Alg+ZnO, GG/Alg+BAC, and GG/Alg+ZnO+BAC, which were subjected to freeze drying. The water absorption of all materials exceeded 4000%, showing excellent liquid absorbability. Burst release of BAC was found at a level of 90% in the first 2 h. In vitro cytotoxicity studies on L929 fibroblasts did not show a toxic effect of extracts from the GG/Alg and GG/Alg+BAC samples, contrary to samples supplemented with ZnO(n). In microbiological studies, the enhanced antibacterial effect of ZnO(n) and BAC was observed in contact with *Staphylococcus aureus* and *Staphylococcus epidermidis* strains. Therefore, GG/Alg+BAC+ZnO is the most promising dressing system for the treatment of infected and exuding wounds.

## 1. Introduction

Polysaccharide-based hydrogels considered for the treatment of wounds, burns, and skin infections are most often obtained from cellulose, chitosan, alginates, and starch [1]. Their advantage is maintaining a moist environment that promotes healing, relieves pain, and reduces the risk of infection. For heavily exuding wounds, dressings capable of absorbing large amounts of fluid are required. These dressings can be produced by freeze drying polysaccharide hydrogels [2]. The resulting porous, spongy microstructure allows for the absorption of exudate, thus cleaning the wound [3]. Particularly promising could be the application of gellan gum (GG), an anionic polysaccharide produced by the bacterium *Sphingomonas elodea*, widely used in food science, tissue engineering and drug delivery [4,5], alone or in combination with other polysaccharides.

The addition of antibiotics, such as bacitracin (BAC), effectively combats the pathogens present in the wound bed [6]. BAC is a polypeptide antibiotic that is routinely used in wound care for its effectiveness against Gram+ bacteria, particularly *Staphylococcus aureus* and *Streptococcus pyogenes*. BAC demonstrates antibacterial activity and minimal systemic absorption, making it suitable for localized application in the form of ointments [7]. It can also be incorporated into dressings to prevent infection in minor cuts and burns [8]. For example, dressings based on bacterial cellulose with the addition of BAC have been proposed in the literature [4,9]. BAC can also be combined with other antibiotics (e.g., amoxicillin) in dressings to increase the antibacterial effect [10]. As a result, BAC remains a common component in over-the-counter and clinical wound dressings, valued for its cost effectiveness and ease of use [8]. However, the growing problem of antimicrobial resistance (AMR), resulting from the excessive use of antibiotics, including topical ones, requires the search for solutions that reduce their dosage while maintaining effectiveness [11]. One such solution is the use of additional modifiers with antimicrobial activity, such as ZnO particles. ZnO primarily targets Gram+ bacteria, damaging their cell wall, generating reactive oxygen species, and releasing Zn^2+^ ions, which disrupt the metabolism of bacterial cells [12].

In this study, we hypothesized that the combination of ZnO particles with BAC in the hydrogel-based dressings can act synergistically, allowing a lower effective antibiotic dose to be administered, thus reducing the concerns of AMR. We considered the application of ZnO particles with two size ranges, micrometric and nanometric, and found that only the latter have antimicrobial activity, particularly against Gram+ bacteria. To this end, we developed highly porous polysaccharide dressings based on GG and sodium alginate (Aln) supplemented with BAC and/or ZnO(n), intended for the treatment of infected and heavily exuding wounds. We analyzed the structure of the obtained materials, and characterized the water absorbability, BAC release, cytotoxicity with L929 fibroblasts, and antimicrobial properties with *Staphylococcus aureus*, *Staphylococcus epidermidis*, and *Streptococcus pyogenes*. To our knowledge, this dressing system has not been reported in the literature so far.

## 2. Results and Discussion

The first part of the present study aimed to characterize nano- and micrometric ZnO, i.e., ZnO(n) and ZnO(m), in an attempt to determine which one has the best properties for infected wound management. In the second part of the work, GG/Alg matrices enriched with selected ZnO(n) and/or BAC were manufactured, and their physicochemical, biological, and bactericidal properties were characterized.

### 2.1. Morphology, Size and Surface Area of ZnO(n) and ZnO(m)

**Figure 1:**
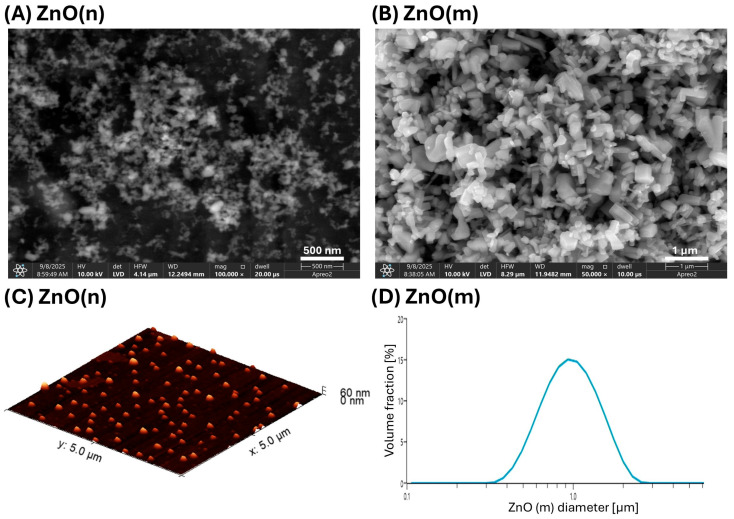
Images of ZnO(n) (**A**) and ZnO(m) (**B**) obtained using scanning electron microscopy at magnification 100,000× and 50,000×, respectively, acquired using LVD—low-vacuum detector; image of ZnO(n) obtained using atomic force microscopy in contact mode, 3D projection (**C**); and volume-based particle size distribution of ZnO(m) obtained from laser diffraction measurements (**D**).

Figure 1A,C show images of ZnO(n) acquired by using scanning electron microscopy (SEM) and atomic force microscopy (AFM), respectively, while Table 1 shows their size measured from AFM pictures and using the Brunauer–Emmett–Teller (BET) relative surface area. ZnO(n) are 26 ± 4 nm in size and have a surface area of 39.16 m^2^/g. It is coherent with work by Mohandas et al. [13], who reported that ZnO particles in the range of 70–120 nm are optimal for infected wound dressings. Considering that particle size significantly affects specific surface area, ion release, and antibacterial activity, independent particle size and BET analysis were essential for reliable material characterization. Figure 1B shows SEM images of ZnO(m), while Figure 1D shows their size distribution obtained from laser diffraction measurement. ZnO(m) are much larger than ZnO(n), 1.02 ± 0.01 µm, and have a much lower relative surface area of 4.56 m^2^/g. The high surface area of ZnO(n) may positively impact the antibacterial activity of the particles, as a large specific surface area can generate more reactive oxygen species (ROS), damaging bacterial cell membranes [14]. It has also been reported that the nanometric dimensions of ZnO also improve its solubility in water and the release of Zn^2+^ ions, which can disrupt the continuity of the bacterial cell membrane [15], making ZnO(n) more effective for infected wound management.

### 2.2. Antimicrobial Activity of ZnO(n) and ZnO(m)

**Figure 2:**
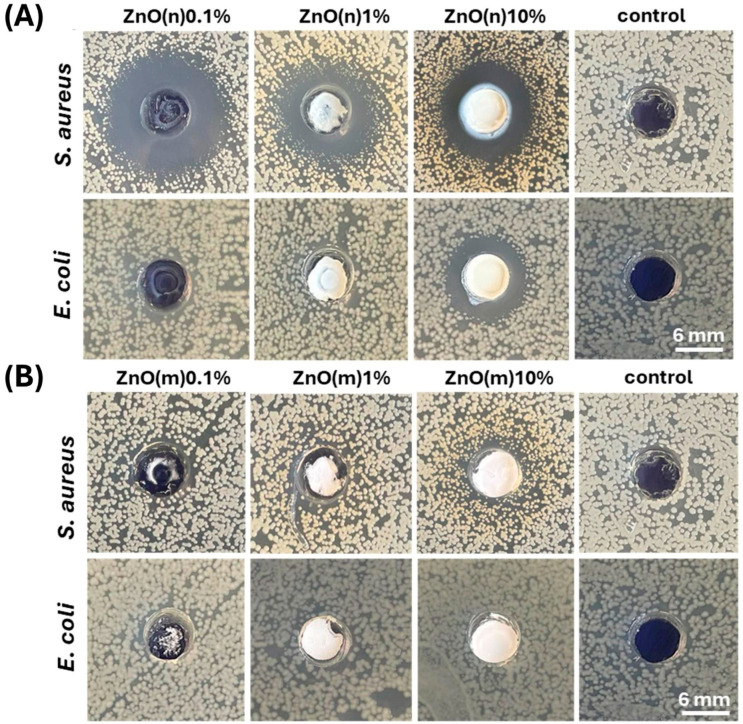
Zones of inhibition (ZOIs) for suspensions of ZnO nanoparticles, ZnO(n) (**A**) and microparticles, ZnO(m) (**B**) at concentration of 0.1%, 1% and 10% on *S. aureus* and *E. coli* strains. Dulbecco’s phosphate-buffered saline, without calcium and magnesium ions (DPBS), acted as control.

Figure 2 shows the zones of inhibition (ZOIs) of the *S. aureus* and *Escherichia coli* strains for suspensions of ZnO(n) and ZnO(m). The results show that ZnO(m) did not produce a visible antibacterial effect in contact with both strains. The ZnO(n) suspensions showed an evident antibacterial effect at all concentrations for the *S. aureus* strain (ZOI: 11–16 mm), and a visible inhibition zone for the *E. coli* strain at a 10% concentration of ZnO(n) (ZOI: 8 mm). The micrometric size of the particles results in a lower solubility of ZnO in water than for the nanoparticles; therefore, the release of Zn^2+^ ions is relatively low. The size of ZnO(m), above 1 µm (Figure 1D, Table 1), also prevents them from penetrating bacterial cells. ZnO(n), due to their small size, sediment slower, allowing for diffusion through the agar. Studies by Prasanna et al. show that ZnO nanoparticles around 30 nm in diameter generate 10 times more OH· and 20 times more H_2_O_2_ than microparticles around 2 µm in diameter [16]. So, we can expect that nanoscale size and high surface area of ZnO(n) allow them to generate higher amounts of ROS, which presumably damage bacterial cell walls more effectively. The higher toxicity of nanoparticles towards *S. aureus* bacteria compared to that of *E. coli* is probably due to differences in the structure of these bacteria’s cells. *S. aureus* (Gram+) bacteria have a thicker cell wall than *E. coli* (Gram-), but they are more porous, making particle penetration easier. *S. aureus* lacks an outer lipid membrane, and the presence of anionic groups on the surface of the cell wall attracts positively charged Zn^2+^ cations [17]. As a result of the antibacterial activity of ZnO(n), this type of particle was used in manufacturing composite samples with GG/Alg samples.

### 2.3. Gross Morphology and Microstructure of GG/Alg Composites

**Figure 3:**
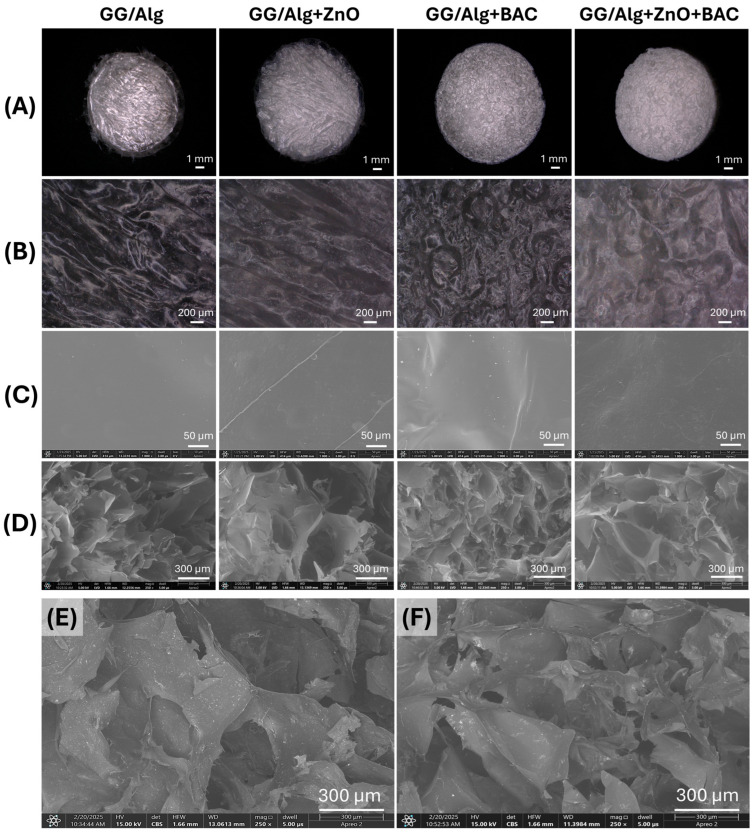
Images of GG/Alg, GG/Alg+ZnO, GG/Alg+BAC and GG/Alg+ZnO+BAC samples obtained using optical digital microscopy: panel (**A**)—magnification 20×, panel (**B**)—magnification 100×; images obtained using scanning electron microscopy: panel (**C**)—surface, magnification 1000×; panel (**D**)—cross section, magnification 250×; acquired using LVD—low-vacuum detector; and pictures of: (**E**)—GG/Alg+ZnO and (**F**)—GG/Alg+ZnO+BAC—cross section, magnification 250×; acquired using CBS—concentring backscatter detector.

Figure 3, panels A and B present optical microscope images of the lyophilized composite samples at magnifications of 20× and 100×, respectively. The surface is rough but nonporous. At 1000× magnification under scanning electron microscopy (SEM), the surface of the samples is relatively smooth (Figure 3, panel C). Figure 3, panel D shows SEM images of the cross section of the samples, which is sponge-like with a very high porosity and a pore size in the range of 300–600 µm. The images clearly depict the sample’s morphology and are acquired using the low-vacuum detector (LVD). These images reveal the three-dimensional microstructure of the samples. Figure 3E,F show SEM images of cross sections of the GG/Alg+ZnO and GG/Alg+ZnO+BAC samples, respectively, acquired using a concentring backscatter (CBS) detector. It is sensitive to differences in atomic number, which allows for chemical contrast—areas of the sample differing in chemical composition are imaged with different contrast. Therefore, despite the less visible topographic details than with the LVD, this detector allowed us to image the ZnO(n) distribution in the sample. Zinc is the heaviest element present in the samples (with an atomic mass of 65.38 u), so bright areas in the CBS detector images indicate the presence of ZnO(n). A homogeneous distribution of zinc oxide is visible within the samples.

### 2.4. Water Absorption and Bacitracin (BAC) Release

**Figure 4:**
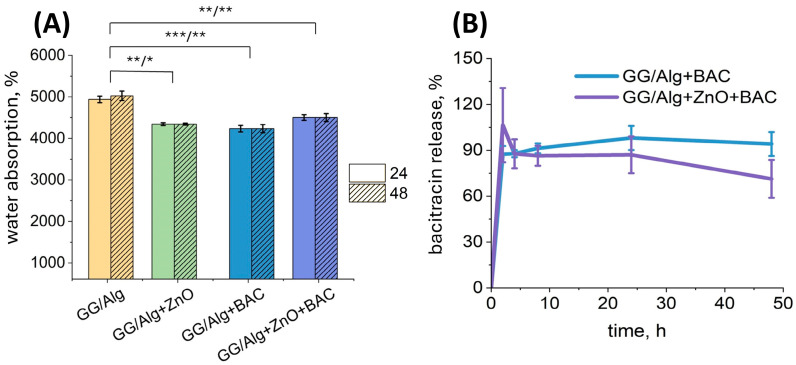
Water absorption rate after 24 h and 48 h of incubation of GG/Alg, GG/Alg+ZnO, GG/Alg+BAC and GG/Alg+ZnO+BAC samples in MilliQ water (**A**) and bacitracin release from GG/Alg+BAC and GG/Alg+ZnO+BAC over 48 h in MilliQ water (**B**). Average from *n* = 3 samples ± SD (standard deviation). Statistically significant differences at * *p* < 0.05, ** *p* < 0.01 and *** *p* < 0.001.

The water absorption of all the samples tested exceeded 4000%, indicating the ability of the composite materials to absorb liquids weighing more than 40 times the dry weight of the sample (Figure 4A). A swelling ratio of 4000% corresponds to a water content exceeding approximately 97% in the fully hydrated state, consistent with highly absorbent alginate-based wound dressings dedicated to bacteria-infected, high exuding wounds, where swelling ratios of up to 5000% have been described by others [18]. There is no significant difference between water absorption values after 24 and 48 h, so the sample absorbs water mainly within the first 24 h, with no increase in water absorption observed after this time. The GG/Alg sample exhibited significantly higher water absorption compared to all other samples, likely due to the absence of modifiers that are released into the liquid environment during the test.

BAC release studies indicate that most drug (90%) is released from samples within the first 2 h (Figure 4B). The fast release of BAC from lyophilized samples is most likely due to their high porosity, which provides a large surface area in contact with water, making it easier for the drug to penetrate the sample and be washed out. Burst release of the drug from samples may be beneficial in the early phase of wound healing to prevent the development of infection, as well as in the event of sudden onset of pain or inflammation to alleviate symptoms quickly. Moreover, the rapid release of the active substance reduces the risk of antimicrobial resistance due to the absence of extended exposure to subinhibitory doses. However, burst release may pose risks, as the rapid release of a large amount of drug over a short period can lead to a toxic effect [19]. It should be emphasized that the results obtained here are a proof of concept, confirming the functionality of the developed dressing prototype. Under in vivo conditions, this process is likely to be more controlled and slower. This is because, during exudate absorption, the dressing gradually swells, leading to a delayed release of the active substance, which is correlated with the amount of exudate absorbed. The clinical performance of such systems depends on balancing rapid initial antibacterial action with controlled subsequent release. Hydrogel wound dressings with adjusted crosslinking have been shown to influence drug release behavior and antibacterial performance, indicating that modulation of network density and bonding can provide fine control over release kinetics [20]. Furthermore, recent studies highlight that mitigating burst release while preserving sustained delivery remains very challenging in the treatment of infected wounds [21].

### 2.5. In Vitro Cytotoxicity

**Figure 5:**
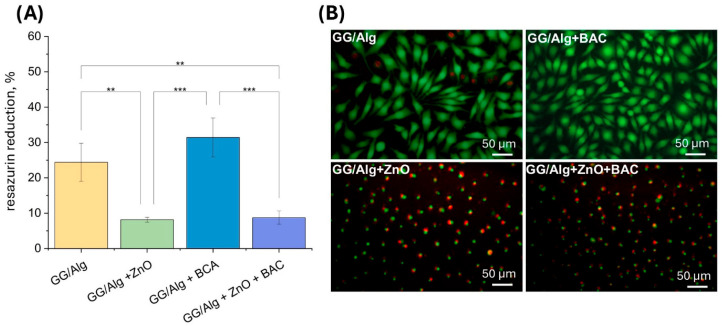
Viability (resazurin reduction AlamarBlue test) (**A**) and live/dead images (fluorescence staining with calcein AM and PI; live cells are stained green while dead cells are stained red) (**B**) of L929 fibroblasts cultured for 24 h in contact with 1% extracts from GG/Alg, GG/Alg+ZnO, GG/Alg+BAC and GG/Alg+ZnO+BAC samples. Average from *n* = 3 samples ± SD (standard deviation). Statistically significant differences at ** *p* < 0.01 and *** *p* < 0.001.

The results of the AlamarBlue test (Figure 5A), which show the metabolic activity of the L929 cells cultured in the extracts, demonstrate a high resazurin reduction of 24.4 ± 5.4% for the GG/Alg control sample and 31.4 ± 5.4% for the GG/Alg+BAC sample. After live–dead fluorescence staining (Figure 5B), cells for the latter samples are mainly stained green, are spindle-shaped, and well spread, indicating that they are alive and that these samples are not cytotoxic. On the other hand, cells cultured in extracts of samples containing ZnO(n) show a reduced viability of 8.2 ± 0.7% for GG/Alg+ZnO and 8.7 ± 1.9% for GG/Alg+ZnO+BAC. After live–dead staining, the green-stained cells are not flattened, but are round and less adherent to the substrate. They are also stained red, which means that they are dying. The biological activity of ZnO(n) is dose-dependent and has been associated with mechanisms including reactive oxygen species (ROS) generation and Zn^2+^ ion release, both of which contribute to oxidative stress and cytotoxic effects in mammalian cells [22]. However, it must be kept in mind that these conditions of cell culture in extracts are very harsh, because of the very fast release of leachable ingredients in the extracts. Under clinical conditions, the release of cytotoxic moieties from the dressing will be slower and correlated with the absorption of the wound exudate.

### 2.6. Antimicrobial Activity

**Figure 6:**
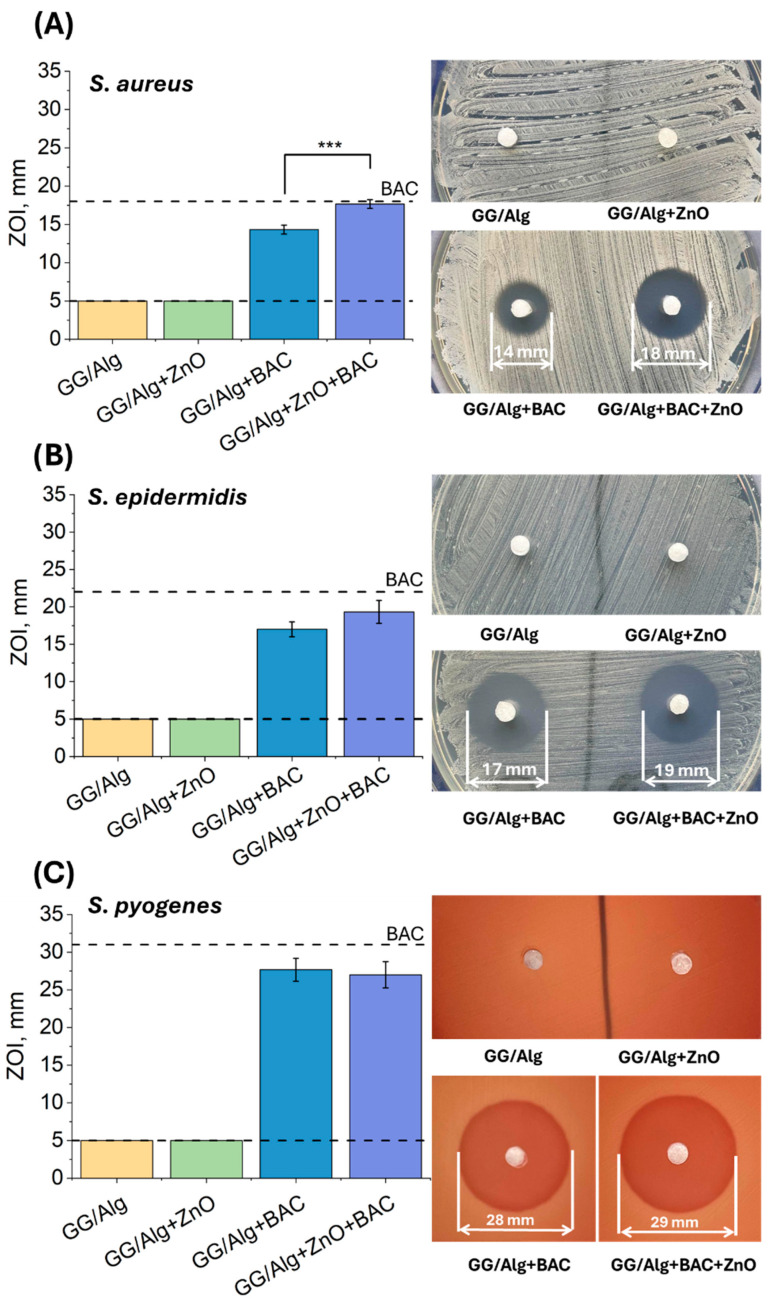
Growth inhibition zones of *S. aureus* (**A**), *S. epidermidis* (**B**), and *S. pyogenes* (**C**) strains in contact with GG/Alg, GG/Alg+ZnO, GG/Alg+BAC and GG/Alg+ZnO+BAC samples. Average from *n* = 3 samples ± SD (standard deviation). Statistically significant difference at *** *p* < 0.001. Lower dashed line shows the diameter of the well, while upper dashed line indicates the inhibition zones for the control sample, i.e., a paper disk impregnated with 10 units of BAC.

The antibacterial properties of the tested samples were assessed against three Gram+ bacteria strains: *S. aureus*, *S. epidermidis*, and *S. pyogenes* (Figure 6). For the *S. aureus* strain (Figure 6A), no antimicrobial activity was observed in the control sample (GG/Alg) or the sample containing ZnO(n) (GG/Alg+ZnO). However, the BAC-containing samples demonstrated significant antibacterial activity: The zones of inhibition (ZOIs) for GG/Alg+BAC were 14.3 ± 0.6 mm, while for GG/Alg+ZnO+BAC they was greater, reaching 17.7 ± 0.6 mm. The larger diameter of the ZOIs for the sample with both modifiers suggests a potential synergistic effect of ZnO(n) and BAC. A similar effect was observed against the *S. epidermidis* strain (Figure 6B): Samples without antibiotics did not show antimicrobial activity. At the same time, samples with BAC have a ZOI of 17.0 ± 1.0 mm for GG/Alg+BAC and 19.3 ± 1.5 mm for GG/Alg+ZnO+BAC. In this case, too, the presence of ZnO(n) enhanced the effect of BAC.

For the *S. pyogenes* strain (Figure 6C), ZnO(n) did not demonstrate antimicrobial activity, but the antibiotic itself was very effective—the zone diameter for the GG/Alg+BAC sample was 27.7 ± 1.5 mm, and for GG/Alg+ZnO+BAC, it was 27.0 ± 1.7 mm. The undetected antimicrobial activity of the composite samples containing only ZnO(n) may be due to the limited diffusion of the nanoparticles in the agar medium or the low concentration in the test environment. In comparison, ZnO(n) suspensions (see Figure 2A) demonstrated antimicrobial activity against *S. aureus*, suggesting significant differences in the mode of action of ZnO(n) in suspension and incorporated into a hydrogel matrix. Although the available literature lacks reports of synergy between ZnO and BAC, there are studies describing the interaction of ZnO with other antibiotics, such as ciprofloxacin and ampicillin (against *S. aureus* and *E. coli*) [23], meropenem (*Pseudomonas aeruginosa*) [24], and colistin (*P. aeruginosa*) [25]. In the case of BAC, the synergistic effect with ZnO may result from the formation of complexes with Zn^2+^ ions, which enhance the antimicrobial effect by inhibiting the growth of Gram+ bacteria and inducing cell lysis [26].

## 3. Conclusions

Antibiotic resistance is a serious issue in modern medicine. One strategy to reduce the amount of antibiotics in medical devices involves the use of antibacterial modifiers that enhance the antimicrobial properties of the material. Advanced dressings designed for infected wounds are often enriched with antibacterial agents such as antibiotics or metal oxide nanoparticles. Microbiological tests confirmed that ZnO(n), unlike ZnO(m), exhibited antibacterial activity—particularly against Gram+ *S. aureus*.

A hydrogel composite based on GG and Alg, and enriched with BAC and ZnO(n), was proposed as a potential wound dressing. Microscopic analysis revealed that the samples were highly porous. Scanning electron microscopy (SEM) with a CBS detector confirmed the homogeneous distribution of ZnO(n) in GG/Alg+ZnO+BAC dressings. Swelling tests showed that the lyophilized samples could absorb over 4000% of their own weight in water, indicating a strong potential for the absorption of wound exudate. A burst release of BAC—approximately 90% within the first two hours—suggests that these dressings could be effective in the early stages of wound healing. In vitro cytotoxicity tests showed that ZnO(n) induced cytotoxic effects on fibroblasts. However, cytotoxicity studies performed on extracts do not mimic clinical situations. In these studies, whole samples were placed in water, which resulted in the rapid release of active ingredients into the extract. In clinical use, a dressing placed on a wound with exudate would release antibacterial agents at a slower rate, which could contribute to a reduction in cytotoxicity.

Tests of lyophilized samples against *S. aureus* and *S. epidermidis* revealed an enhanced effect of ZnO(n) on the antibacterial activity of bacitracin in the case of the GG/Alg+ZnO+BAC sample. Interestingly, GG/Alg+ZnO alone did not exhibit any antibacterial activity. Based on these results, the GG/Alg+ZnO+BAC sample demonstrated the most promising antibacterial properties, especially against *S. aureus* and *S. epidermidis*, which cause the most common wound infections. The results obtained allowed us to assess the antibacterial effectiveness of individual modifiers and show the preliminary evidence of cooperative or additive antibacterial interaction between ZnO and BAC, which may provide the basis for the further development of antibacterial dressings. However, due to the cytotoxicity of the extract to fibroblasts, further optimization is necessary to adjust the concentration of ZnO(n) to ensure the enhanced antibacterial effect while minimizing toxicity. It is also suggested to evaluate obtained samples in more relevant in vitro and in vivo models, resembling clinical conditions of infected and exuding wounds.

## 4. Materials and Methods

### 4.1. Materials

Micrometric zinc oxide (ZnO(m)) was provided by ZM Silesia SA, Oława, Poland. Nanometric zinc oxide (ZnO(n)), sodium dodecyl sulfate (SDS), poly(vinyl alcohol) (PVA, Mowiol 4-88, MW 31 kDa), gellan gum (GG, Gelzan^TM^, MW 200–300 kDa), sodium alginate from brown algae (Alg), CaCl_2_·6H_2_O, bacitracin A (BAC), bicinchoninic acid, copper (II) sulfate pentahydrate (CuSO_4_·5H_2_O), phosphate-buffered saline (PBS), AlamarBlue reagent, calcein AM and propidium iodide (PI) came from Sigma-Aldrich, St. Louis, MO, USA. Ethanol was provided by POCH (Gliwice, Poland). Dulbecco’s modified Eagle’s medium (DMEM), fetal bovine serum (FBS), penicillin/streptomycin and Dulbecco’s phosphate-buffered saline, without calcium and magnesium ions (DPBS) were from PAN-Biotech, Aidenbach, Germany. Muller–Hilton agar and Columbia agar with 5% sheep blood were from Becton Dickinson, Franklin Lakes, NJ, USA. L929 fibroblasts came from the European Collection of Cell Cultures, UK. Bacteria strains *Staphylococcus aureus* ATCC^®^ 25923™, *Escherichia coli* ATCC 25922™, *Staphylococcus epidermidis* ATCC^®^ 700296™ and *Streptococcus pyogenes* ATCC^®^ 12384™ were from American Type Culture Collection, Manassas, VA, USA.

### 4.2. Atomic Force Microscopy (AFM)

For measuring ZnO(n) size using an AFM, a suspension of 0.02% *w*/*v* ZnO(n) was prepared in MilliQ water. The suspension was ultrasonicated for 15 min. One drop of the prepared suspension was placed on the coverslip and placed in a 37 °C incubator until the water evaporated. The prepared sample was placed under an AFM (Explorer, Thermomicroscopes, Sunnyvale, CA, USA) and observed in contact mode. The resulting image was processed, and the ZnO(n) diameters were measured using the Gwyddion AFM image visualization program (Version 3.1).

### 4.3. Laser Diffraction Measurements

The diameter of ZnO(m) was measured using a laser diffraction technique (MasterSizer 3000E; Malvern Instruments; Hydro SV, Worcestershire, UK). A 0.1% *w*/*v* suspension of ZnO(m) was prepared in MilliQ water of 0.25% *w*/*v* SDS and 0.2% *w*/*v* PVA and placed in an ultrasonic bath for 30 min to minimize particle agglomeration before the measurements. Measurements were made at an obscuration level of 5–10% with a stirrer speed of 500 rpm. The measurement was performed in triplicate. The results were reported as volume-based particle size distribution and volume-weighted mean diameter.

### 4.4. Surface Area

The surface area of ZnO(n) and ZnO(m) was determined using the BET method with an adsorption analyzer (ASAP 2020 Plus, Malvern Panalytical, Norcross, GA, USA). The samples were firstly degassed at 120 °C for 24 h before the analysis and the N_2_ adsorption was at −196 °C.

### 4.5. Preparation of Composites with ZnO and BAC

**Figure 7:**
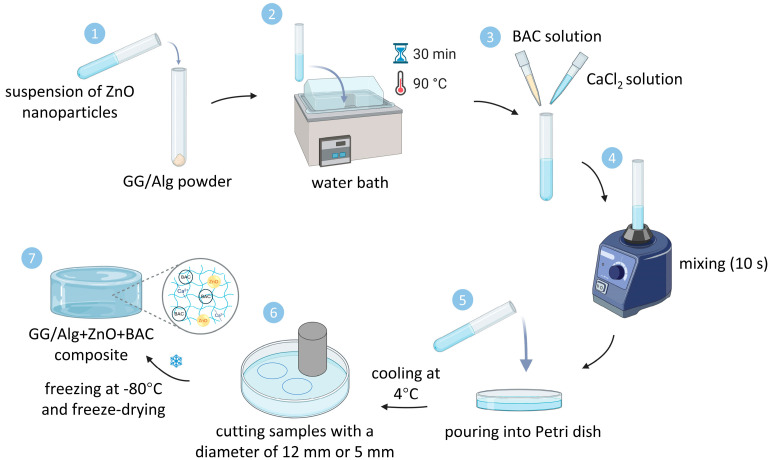
Schematic representation of preparation of GG/Alg+ZnO+BAC samples.

GG/Alg samples were prepared by dissolving in 8 mL of MilliQ water GG and Alg powders in a weight ratio of 10:1 for 30 min in a water bath at 90 °C. Before this step, ZnO(n) was suspended in MilliQ water and vortexed for 10 min for the samples with ZnO. The temperature was then lowered to 70 °C. A solution of BAC in 1 mL of MilliQ water was added for the samples with BAC. Then, 1 mL of 0.6% CaCl_2_·6H_2_O solution heated to 70 °C was added and vortexed for 10 s. After that, the samples were poured into a 9 cm diameter Petri dish with spacers (4 mm high) and covered with a glass slide. The composition of the manufactured samples without and with modifiers is presented in Table 2, while the manufacturing method is shown in Figure 7. For the experiments, the samples with a diameter of 12 mm were cut, frozen, and freeze-dried (Alpha 1–2, Martin Christ, Osterode am Harz, Germany). Only for microbiological tests, the diameter of the samples was 5 mm.

### 4.6. Optical Microscopy

The freeze-dried samples were observed under an optical digital microscope (Keyence VHX 7000, Mechelen, Belgium) at magnifications of 20× and 100×.

### 4.7. Scanning Electron Microscopy (SEM)

The ZnO particles were observed using SEM. Before SEM observations, ZnO(n) and ZnO(m) were suspended in ethanol and placed in an ultrasonic bath for 15 min. Then 20 µL of each suspension was placed on a metal holder and left to dry at 37 °C. The samples were observed under a SEM (ThermoFisher Scientific Apreo 2, Waltham, MA, USA) at 10 kV and a magnification of 100,000× and 50,000× using a low-vacuum detector (LVD).

For SEM imaging of the composites, two samples of each type were prepared: one for surface imaging and one for cross-sectional imaging. Samples were carbon-sputtered. For external surfaces, observations were performed at 1000×, while cross-sectional imaging was performed at 250× using LVD. In the case of cross sections of samples containing ZnO (GG/Alg+ZnO, GG/Alg+BAC+ZnO), imaging was also performed using a concentring backscatter (CBS) detector.

### 4.8. Swelling Capacity

The lyophilized samples (*n* = 3 of each type) were weighed and then immersed in 2 mL of MilliQ water each. After 24 h and 48 h, the samples were collected, and carefully wiped with a wet Kimtech tissue (Kimwipes, Kimberly-Clark, Dallas, TX, USA) to remove excess water, but avoiding removing water from the pores of the sample.

The swelling of the samples was calculated from Formula (1):

(1) $$\%Swelling=\frac{M_{w}-M_{d}}{M_{d}}\times 100\%$$

where $M_{w}$—mass of the wet sample, $M_{d}$—mass of the dry sample.

### 4.9. Bacitracin (BAC) Release Studies

The release of BAC was examined from lyophilized GG/Alg+BAC and GG/Alg+ZnO+BAC samples. The samples were weighed and then placed in Falcon tubes with 5 mL of PBS solution. The tubes were placed in an incubator at 37 °C. After 2, 4, 8, 24, and 48 h, 0.5 mL of the solution was withdrawn from the tubes into Eppendorf tubes for BAC determinations, each time topping the collected fluid with PBS. BAC was determined using the bicinchionic acid assay (BCA). To determine the BAC concentration, a calibration curve was prepared. BAC solutions of the following concentrations in PBS were prepared: 750, 500, 400, 300, 200, 100, 50, 20, and 0 µg/mL. Subsequently, a BCA reagent was prepared by mixing BCA with a 4% CuSO_4_·5H_2_O solution at a 50:1 volume ratio. A total of 50 µL of each calibration solution and 50 µL of test samples containing released BAC were pipetted in triplicate into a transparent 96-well plate. Next, 100 µL of the BCA reagent was then added to each well. The absorbance of each sample was measured after 15 min of incubation using a FLUOstar Omega (BMG Labtech, Ortenberg, Germany) reader at 562 nm. On the basis of the absorbance data, a calibration curve was prepared, and the BAC concentration released over time was determined.

### 4.10. In Vitro Cytotoxicity

In vitro cytotoxicity was evaluated using L929 fibroblasts cultured in DMEM supplemented with 10% FBS and a 1% mixture of penicillin and streptomycin. Cells were cultured at 37 °C in a 5% CO_2_ atmosphere. A total of 200 µL of cell suspension (50,000 cells/mL) was added to each well in the 96-well plate (10,000 cells per well). The cells were then left to adhere to the bottom of the wells. Simultaneously, 1% extracts of lyophilized samples were prepared by incubation of 20 mg of samples in 2 mL of DMEM for 24 h. Before extraction, the samples were irradiated under UV light (15 min on each side). After 24 h, the medium was aspirated and replaced with extracts. Cells were incubated with 1% *w*/*v* extracts of the tested materials, while control cells were cultured in standard culture medium without extracts (*n* = 3). The AlamarBlue assay was used to assess cell viability. After 24 h, the extracts in each well were replaced with 150 µL of a 5% AlamarBlue reagent solution in DMEM. After 3 h of incubation, 100 μL aliquots were transferred to the wells of a 96-well black plate and the fluorescence intensity was measured at λex = 530 nm and λem = 590 nm using the FluoroSTAR Omega reader. The following Formula (2) was used to determine the percentage of resazurin reduction:

(2) $$\%Resazurin\;reduction=\frac{F_{x}-F_{0\%}}{F_{100\%}-F_{0\%}}\times 100\%$$

where $F_{x}$—fluorescence of the sample, $F_{0\%}$—fluorescence of the AlamarBlue solution after 3 h incubation without cells, $F_{100\%}$—fluorescence of the AlamarBlue reagent reduced completely in an autoclave.

Live/dead analysis of the cells was performed by replacing the extracts with 100 µL of staining solution containing 0.1% calcein AM and 0.1% propidium iodide (PI) prepared in DPBS. After incubation for 20 min at 37 °C, cells were observed under a fluorescence microscope (Axiovert 40 CFL, Carl Zeiss, Oberkochen, Germany).

### 4.11. Antimicrobial Activity

For microbiological studies of ZnO(n) and ZnO(m), suspensions were prepared in DPBS at concentrations of 0.1%, 1%, and 10% *w*/*v*. Antibacterial activity was tested on *S. aureus* and *E. coli* strains. Bacterial suspensions with a density of 0.5 McFarland were prepared and spread on a Muller–Hilton agar plate (Becton Dickinson, Franklin Lakes, NJ, USA) using a disposable swab. The wells were cut on the agar using a 5 mm sterile punch. A total of 100 µL of ZnO suspensions were added to the wells (three replicates for each concentration); 100 µL of DPBS solution was used as a control. The dishes were placed at 4 °C for 4 h to allow initial diffusion of antibacterial moieties in the agar. The dishes were then incubated in a 37 °C incubator for 18 h. After this time, the diameters of the resulting ZOIs were measured.

For microbiological testing of the lyophilized hydrogels, samples measuring 5 mm in diameter were used. Suspensions of three bacterial strains were prepared: *S. aureus*, *S. epidermidis*, and *S. pyogenes* in a DPBS solution with a density of 0.5 McFarland. Muller–Hilton agar was used for *S. aureus* and *S. epidermidis* strains, and Columbia agar with 5% sheep blood was used for *S. pyogenes* strains. Bacteria were transferred to the agar medium using a disposable swab. One control sample (a 5 mm diameter well with DPBS solution) and a 6 mm diameter diagnostic paper disk impregnated with 10 units of BAC were placed on the prepared agar plates, along with samples of GG/Alg, GG/Alg+ZnO, GG/Alg+BAC, and GG/Alg+ZnO+BAC (*n* = 3). The plates with samples were left in the refrigerator for 4 h and then placed in an incubator at 37 °C for 24 h. After 24 h, the diameters of the ZOIs were measured.

### 4.12. Statistics

Statistical analysis was performed using one-way analysis of variance (one-way ANOVA) followed by Tuckey’s post hoc test using OriginLab2023 software. Differences were considered significant when *p* < 0.05. The results are presented as mean ± standard deviation (SD). Statistically significant differences * *p* < 0.05, ** *p* < 0.01 and *** *p* < 0.001 are shown.

## Figures and Tables

**Table 1:** Average diameters and BET surface area of ZnO(n) and ZnO(m) particles.

	Diameter, nm/µm	BET Surface Area, m^2^/g
ZnO(n)	26 ± 4 nm *	39.16
ZnO(m)	1.02 ± 0.01 µm **	4.56

*—average diameter obtained from AFM images; **—volume-weighted mean diameter obtained from laser diffraction measurements.

**Table 2:** Composition of hydrogel samples: amount of ingredients per 10 mL MilliQ water.

Samples	Ingredients, mg			
	GG	Alg	ZnO(n)	BAC
GG/Alg	182	18	-	-
GG/Alg+ZnO	182	18	10	-
GG/Alg+BAC	182	18	-	10
GG/Alg+ZnO+BAC	182	18	10	10

## Data Availability

The raw data supporting the conclusions of this article will be made available by the authors on request.
